# A novel bidirectional positive-feedback loop between Wnt–β-catenin and EGFR–ERK plays a role in context-specific modulation of epithelial tissue regeneration

**DOI:** 10.1242/jcs.150888

**Published:** 2014-07-01

**Authors:** Nikolaos T. Georgopoulos, Lisa A. Kirkwood, Jennifer Southgate

**Affiliations:** 1 Jack Birch Unit for Molecular Carcinogenesis, Department of Biology, University of York, York YO10 5DD, UK; 2 Department of Biological Sciences, School of Applied Sciences, University of Huddersfield, Huddersfield HD1 3DH, UK

**Keywords:** Wnt, β-catenin, EGFR, ERK, AKT, Crosstalk

## Abstract

By operating as both a subunit of the cadherin complex and a key component of Wnt signalling, β-catenin acts as the lynchpin between cell–cell contact and transcriptional regulation of proliferation, coordinating epithelial tissue homeostasis and regeneration. The integration of multiple growth-regulatory inputs with β-catenin signalling has been observed in cancer-derived cells, yet the existence of pathway crosstalk in normal cells is unknown. Using a highly regenerative normal human epithelial culture system that displays contact inhibition, we demonstrate that the receptor tyrosine kinase (RTK)-driven MAPK and Wnt–β-catenin signalling axes form a bidirectional positive-feedback loop to drive cellular proliferation. We show that β-catenin both drives and is regulated by proliferative signalling cues, and its downregulation coincides with the switch from proliferation to contact-inhibited quiescence. We reveal a novel contextual interrelationship whereby positive and negative feedback between three major signalling pathways – EGFR–ERK, PI3K–AKT and Wnt–β-catenin – enable autocrine-regulated tissue homeostasis as an emergent property of physical interactions between cells. Our work has direct implications for normal epithelial tissue homeostasis and provides insight as to how dysregulation of these pathways could drive excessive and sustained cellular growth in disease.

## INTRODUCTION

β-catenin is a multifunctional signalling protein that, through regulation of its stability and intracellular localisation, plays a central role in epithelial tissue homeostasis. Downstream of Wnt signalling, β-catenin that has translocated to the nucleus functions indirectly as a transcription factor by modulating the transcriptional activity of transcription factors of the T-cell factor/lymphoid enhancer factor (TCF/LEF) family to promote cell proliferation (Nelson and Nusse, 2004). β-catenin also functions as a structural component of adherens junctions, where its association with E-cadherin stabilises the multi-protein adherens complex at the plasma membrane (Cavallaro and Christofori, 2004). These two properties of β-catenin place it at the interface between cell–cell adhesion and the control of cellular proliferation, as the ability of E-cadherin to sequester β-catenin at the cell membrane can influence the signalling capacity of β-catenin and modulate TCF/LEF transcriptional activity (Nelson and Nusse, 2004).

In the absence of Wnt ligand, β-catenin is phosphorylated by glycogen synthase kinase 3β (GSK3β), a component of the protein ‘destruction’ complex. This complex also contains the scaffold protein axin and the adenomatous polyposis coli (APC) protein. The destruction complex is responsible for the ubiquitylation and subsequent proteasomal degradation of β-catenin. Upon the binding of Wnt ligand to Frizzled (Fzd) receptors, an intracellular signalling cascade triggers the release of β-catenin from the destruction complex for subsequent trafficking to the nucleus or cell membrane (Clevers and Nusse, 2012).

A further layer of complexity relates to the interaction of β-catenin with receptor tyrosine kinase (RTK) and the mitogen-activated protein kinase (MAPK) signalling pathways (Bikkavilli and Malbon, 2009). Wnt ligand is reported to trigger proliferation through extracellular signal-related kinases (ERKs, the classical MAPKs) in fibroblasts (Yun et al., 2005), and β-catenin can induce epidermal growth factor receptor (EGFR) gene expression in hepatocellular (Latasa et al., 2012) and prostate carcinoma (Guturi et al., 2012) cells. By contrast, a number of mitogenic factors, such as EGF, have been reported to induce β-catenin signalling (Ding et al., 2005; Lu et al., 2003; Morali et al., 2001; Müller et al., 2002); EGFR can drive protein kinase B (AKT)–β-catenin-mediated maintenance of nasopharyngeal cancer stem cells (Ma et al., 2013), and various RTKs have been reported to activate canonical Wnt–β-catenin signalling in murine chondrocytes (Krejci et al., 2012). Thus, β-catenin has the capacity to crosstalk with and modulate the function of MAPK pathways to influence cell proliferation; conversely, RTKs can regulate cell growth through β-catenin signalling. In most previous studies, established cell lines derived from carcinomas or non-epithelial (predominantly mesenchymal) origins have been investigated and, hence, the importance of crosstalk between signal transduction pathways in normal epithelial tissue homeostasis and regeneration is unknown.

Here, we have employed a well-characterised normal human urothelial (NHU) cell culture system, in which tissue-isolated cells are maintained as multiple finite (non-immortalised) cell lines *in vitro*. In low-Ca^2+^ (0.09 mM) serum-free medium, NHU cells adopt a highly proliferative phenotype driven by autocrine signalling, with amphiregulin recognised as the key EGFR-activating ligand (Varley et al., 2005). NHU cells exhibit a basal phenotype and do not show spontaneous differentiation at confluence (Lobban et al., 1998; Southgate et al., 1994), although, unlike immortalised human urothelial cells, the capacity for differentiation is retained (Georgopoulos et al., 2011). A second autocrine pathway has been identified in low-density NHU cell cultures maintained in near-physiological concentrations of exogenous Ca^2+^ (2 mM), wherein proliferation is promoted in juxtaposed cells that form stable intercellular junctions. This proliferation pathway is mediated by phospho-activation of AKT and is attenuated by PI3K antagonists or by functional inactivation of E-cadherin – either of which results in derepression of the EGFR–ERK pathway and activation of β-catenin–TCF signalling (Georgopoulos et al., 2010). Our aim here was to elucidate the interactions between EGFR–ERK and Wnt–β-catenin signalling in regulating normal epithelial cell proliferation.

## RESULTS

### Gene expression analysis of Wnt–β-catenin pathway components in proliferating versus quiescent NHU cells

Gene arrays representing proliferating (24 hour) NHU cell cultures were analysed for the expression of Wnt pathway-related transcripts, and this analysis revealed that all the components necessary for a functional Wnt cascade were expressed. Seven Frizzled receptors (Fzd2, Fzd3, Fzd4, Fzd5, Fzd6, Fzd7 and Fzd10) that have been implicated in transducing the canonical Wnt signal were present, as were the transcripts of four Wnt ligands (Wnt3, Wnt5a, Wnt6 and Wnt7a). Transcripts for several downstream targets of Wnt signalling were also expressed by proliferative cultures, including Axin2 (a key target of Wnt signalling), survivin (BIRC5), Twist, SKP2 and cyclin D1. Archetypal extracellular Wnt antagonists, including soluble Fzd-related protein (sFrp) and Wif1, were not transcribed by proliferative cultures. A summary of the results from the expression arrays (and a comparison with results obtained from contact-inhibited quiescent cultures), along with an independent verification of the key findings by RT-PCR, is shown in supplementary material Fig. S1.

### Expression of active β-catenin and pharmacological activation of the canonical Wnt signalling pathway in proliferating NHU cells

**Fig. 1.**
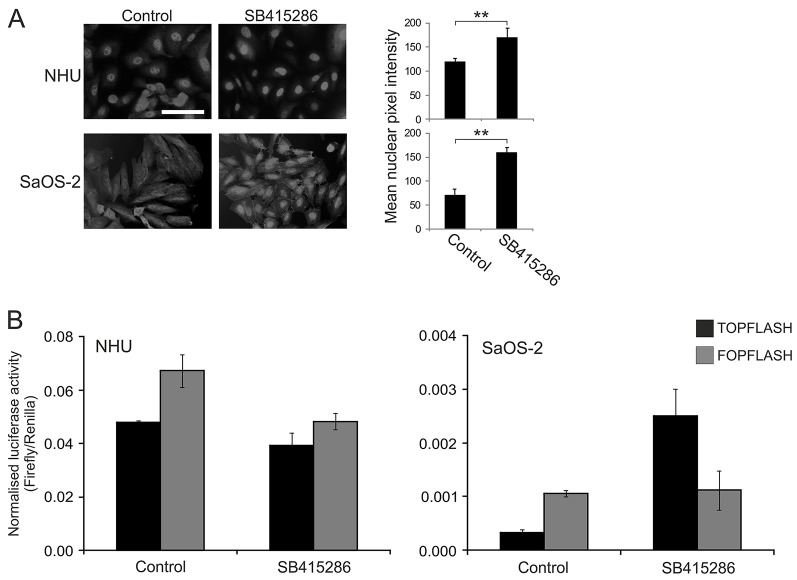
**The effect of Wnt–β-catenin signalling activation by pharmacological inhibition of GSK3β in NHU and SaOS-2 cells.** (A) Left, active β-catenin protein expression was detected by indirect immunofluorescence microscopy in NHU (upper panels) and SaOS-2 cells (lower panels) after 24-hours of culture in medium supplemented with 10 µM GSK3β inhibitor (SB415286). Treatment with solvent alone [0.1% (v/v) DMSO] served as a negative control. The results are representative of at least three independent experiments. Scale bar: 50 µm. Right, nuclear translocation of active β-catenin in SB415286-treated and control cells was also quantified as described in Materials and Methods. The data show the mean nuclear pixel intensity (±s.d.) from independent randomly selected β-catenin-labelled NHU and SaOS-2 cells (*n* = 20); **P<0.01. (B) NHU (left) and SaOS-2 cells (right) were transfected with either TOPFLASH or FOPFLASH plasmid along with pRL-CMV vector as a transfection control. At 24 hours post-transfection, the medium was changed to include 10 µM SB415286 or 0.1% (v/v) DMSO (vehicle control) for an additional 24 hours. Subsequently, dual luciferase assays were performed. Data show the mean±s.d. of firefly luciferase activity for three or four technical replicates following normalisation to the transfection control (Renilla) and are representative of at least three independent experiments.

In low-density culture, NHU cells homogeneously expressed nuclear-restricted active β-catenin, as shown using an antibody that recognised the Ser37 and Thr41 dephosphorylated form of β-catenin. This contrasted with growing cultures of the well-characterised Wnt-responsive SaOS-2 osteosarcoma cell line (Ueda et al., 1997), which exhibited a diffuse cytoplasmic labelling pattern with limited nuclear localisation (Fig. 1A).

The highly specific GSK3β antagonist SB415286 (Meijer et al., 2004) was used to inactivate the destruction complex and trigger canonical Wnt signalling in a ligand-independent manner. Following treatment with SB415286, SaOS-2 cells showed activation and translocation of β-catenin to the nucleus, with some active β-catenin also present at intercellular contacts. By contrast, NHU cells, which already exhibited a high level of nuclear active β-catenin in control cultures, displayed only a modest increase in nuclear labelling after treatment with SB415286 (Fig. 1A). These findings indicate that proliferative NHU cells have an activated Wnt–β-catenin pathway, with a pool of active β-catenin present in the nucleus.

TCF promoter activity was assessed using the TOPFLASH/FOPFLASH luciferase reporter assay. SaOS-2 cells showed a significant increase in luciferase expression when treated with SB415286 (Fig. 1B); normalised TOPFLASH reporter activity after SB415286 treatment was 7.5-fold higher (*P*<0.001) than that of a vehicle-control-treated sample (Fig. 1B), confirming the functionality of the pharmacological activator and its ability to mimic active Wnt signalling. By contrast, there was no significant change (*P*>0.05) in TOPFLASH luciferase activity observed in NHU cells after SB415286 treatment (Fig. 1B).

The FOPFLASH control reporter provided a normalisation control, as it showed constant activity in NHU cells under different culture conditions, although it tended to give higher readouts than were achieved with TOPFLASH; similar observations were also made with untreated SaOS-2 cells (Fig. 1B) and have been reported in other epithelial cells (Papkoff and Aikawa, 1998). TOPFLASH and FOPFLASH are engineered from the same vector backbone and the only difference is the presence of a series of tandem LEF1/TCF-binding sites within TOPFLASH that are mutated in FOPFLASH. However, also extant within the promoter inserts of both vectors are a plethora of other transcription-factor-binding sites, including those of nuclear receptor sub-family 2, GATA and PPARγ, which account for the high basal activity seen.

### Blockade of EGFR–ERK and PI3K–AKT pathways reveals positive feedback between EGFR–ERK and β-catenin signalling

**Fig. 2.**
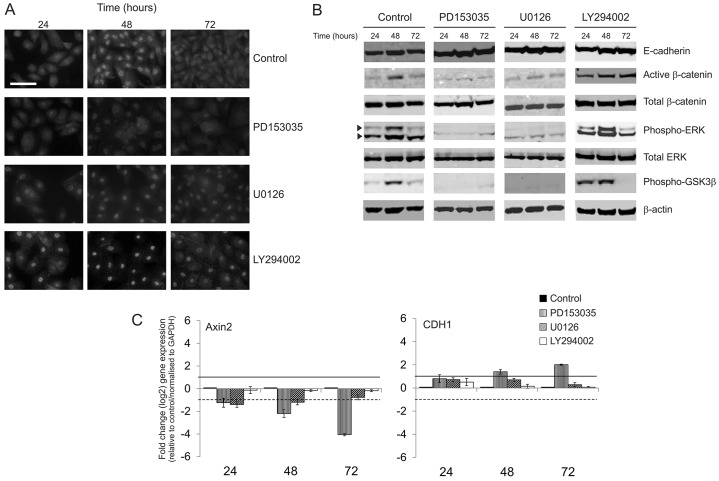
**Effect of inhibition of EGFR, MEK–ERK and PI3K–AKT on the Wnt–β-catenin signalling pathway in NHU cells.** (A) NHU cells were cultured for 24, 48 and 72 hours post-seeding in standard culture medium (Control) or medium containing 1 µM EGFR inhibitor (PD153035), 5 µM MEK–ERK inhibitor (U0126) or 5 µM PI3K–AKT inhibitor (LY294002). These parallel cultures were then immunolabelled to detect the expression of active β-catenin. Results are representative of at least three independent experiments. Scale bar: 50 µM. (B) NHU cells were cultured as described for A, before whole-cell lysates were prepared and the expression of E-cadherin, β-catenin (active and total), ERK (phospho- and total) and phospho-GSK3β (serine 9) was assessed by western blotting. For phospho-ERK, detection of both ERK1 (upper band) and ERK2 (lower band) isoforms of molecular mass 44 and 42 kDa, respectively, is indicated by arrowheads. Detection of β-actin served as a loading control. Results are representative of experiments with two NHU lines. (C) NHU cells were cultured as described for A, total RNA was isolated, cDNA was prepared and quantitative real-time RT-PCR was performed for the direct downstream β-catenin–TCF target Axin2 (left). E-cadherin (CDH1) mRNA expression was also quantified (right). Data were initially normalised to GAPDH house-keeping controls and then expressed as fold change relative to solvent-balanced controls. Data represent the log2 mean expression (±s.d.) of three technical replicates. Solid and dotted lines represent twofold upregulation and twofold downregulation, respectively.

In NHU cell cultures grown under standard culture conditions either with or without exogenous EGF, the expression of active β-catenin fluctuated over time. At a seeding density of 2.5×10^4^ cells/cm^2^, nuclear labelling for active β-catenin was most intense at 48 hours post-seeding, after which it was seen to decrease as cells reached confluence (Fig. 2A, Control; supplementary material Fig. S2). Western blotting results from parallel cultures corroborated the immunofluorescence microscopy findings, with active β-catenin expression peaking at 48 hours of culture (Fig. 2B). In parallel, the expression of phosphorylated ERK1/2 (phospho-ERK) also peaked at the 48-hour time-point, indicating a reciprocal pattern of expression for β-catenin and phospho-ERK. To determine whether β-catenin activation was due to a change in the activity of the destruction complex, we probed for the inactive form of GSK3β, which is phosphorylated at Ser9 (da Costa et al., 1999). Our results revealed that the increase in active β-catenin expression seen at 48 hours post-seeding was accompanied by an increase in the amount of inactive phospho-GSK3β (Fig. 2B).

Functional inactivation of EGFR by the pharmacological inhibitor PD153035 blocked the expression of nuclear active β-catenin (Fig. 2A,B). This was accompanied by loss of induction of phospho-GSK3β (Fig. 2B). We have previously shown that EGFR signalling in proliferative NHU cells is predominantly relayed across the MEK–ERK intracellular signalling axis (MacLaine et al., 2008; Varley et al., 2005). Functional ERK blockade by the MEK inhibitor U0126 caused marked attenuation of active β-catenin expression and inhibited the induction of phospho-GSK3β (Fig. 2B). By contrast, functional blockade of PI3K–AKT signalling with LY294002 caused a marked increase in the amount of nuclear active β-catenin at all time-points (Fig. 2A). In this latter state, some cells showed membrane-localised β-catenin, which was not evident in the control cultures (Fig. 2A). Notably, phospho-GSK3β expression was dramatically induced in PI3K–AKT-blocked cells at both the 24- and 48-hour time-points, yet it was diminished by 72 hours (Fig. 2B). Moreover, PI3K–AKT inhibition resulted in a substantial elevation in the levels of phospho-ERK.

To confirm the functionality of β-catenin activation and the downstream effects of RTK signalling blockade, we assessed the expression of Axin2 as a direct target of β-catenin–TCF transcription. The expression of Axin2 was significantly downregulated (by greater than twofold) after treatment with the EGFR inhibitor PD153035 for 24, 48 and 72 hours in culture (Fig. 2C), implying that inhibition of EGFR reduces TCF-mediated transcription. Treatment with the ERK inhibitor (U0126) also reduced the expression of Axin2 at the 24- and 48-hour time-points and, to a lesser extent, at the 72-hour time-point (Fig. 2C). By contrast, PI3K–AKT inhibition (with LY294002) did not alter Axin2 transcription (Fig. 2C). Similar observations were made for another β-catenin–TCF transcriptional target, c-Myc (not shown). We also examined the expression of E-cadherin (CDH1) mRNA, a repressed indirect target of β-catenin–TCF signalling (due to repression by the Wnt target Twist). In contrast to Axin2, CDH1 mRNA expression was significantly higher in EGFR-blocked cultures (Fig. 2C), an observation that mirrored our findings at the level of E-cadherin protein expression (Fig. 2B). Inhibition of neither ERK nor PI3K–AKT affected the expression of the CDH1 transcript (Fig. 2C).

### Blockade of EGFR reveals the capacity for proliferative Wnt signalling in NHU cells

**Fig. 3.**
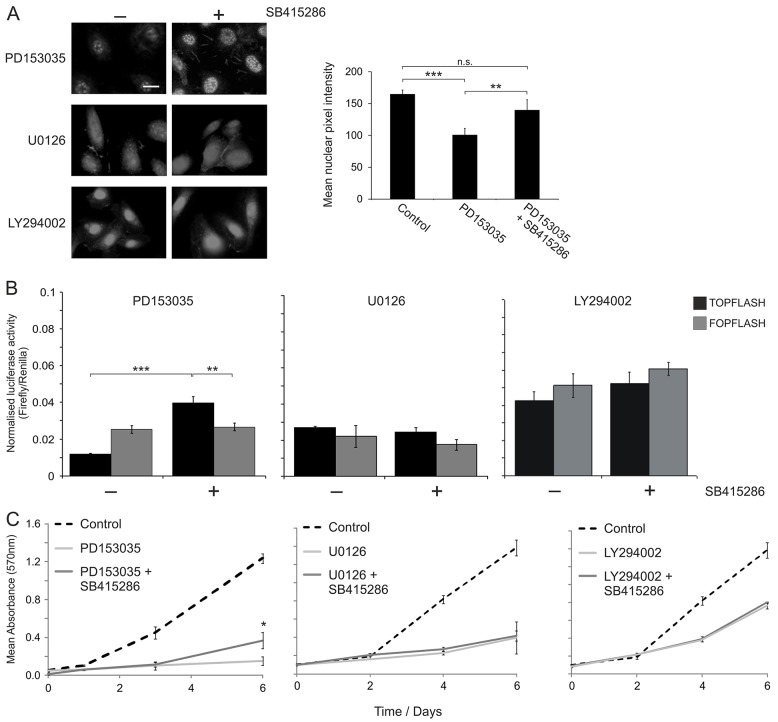
**Pharmacological activation of Wnt–β-catenin signalling in NHU cells treated with EGFR, ERK and AKT inhibitors: effects on β-catenin localisation, TCF transcriptional activity and cell growth.** (A) Left, NHU cells were pre-treated for 24 hours with medium supplemented with 1 µM EGFR inhibitor (PD153035), 5 µM MEK–ERK inhibitor (U0126) or 5 µM PI3K–AKT inhibitor (LY294002). Thereafter, the culture medium was replenished and supplemented with 10 µM GSK3β inhibitor (SB515286) or solvent [0.1% (v/v) DMSO] alone (Control) in the continued presence of signalling inhibitors for a further 24 hours. Cells were then immunolabelled to detect expression of active β-catenin. Results are representative of at least three independent experiments. Scale bar: 20 µM. Right, nuclear translocation of active β-catenin in control cells (micrographs not shown) and PD153035-treated NHU cells in the absence (PD153035) or presence (PD153035 + SB415286) of 10 µM GSK3β inhibitor was also quantified (as described in Fig. 1). Data represent the mean nuclear pixel intensity (±s.d.) from independent randomly selected β-catenin-positive cells (*n* = 20). (B) NHU cells were transfected with either TOPFLASH or FOPFLASH plasmid along with pRL-CMV vector as a transfection control. At 16 hours post-transfection, cells were pre-treated with 1 µM EGFR inhibitor (PD153035), 5 µM MEK–ERK inhibitor (U0126) or 5 µM PI3K–AKT inhibitor (LY294002) for 24 hours. Cells were then incubated for a further 24 hours with 10 µM GSK3β inhibitor (SB415286) in the continued presence of signalling inhibitors. Subsequently, dual luciferase reporter assays were performed. Data show the mean (±s.d.) firefly luciferase activity [three technical replicates following normalisation to the transfection control (Renilla), at least three independent experiments]. **P<0.01; *** *P*<0.001; n.s., non-significant. (C) NHU cells were seeded and allowed to attach overnight. On day 0, cells were cultured in medium supplemented with 1 µM EGFR inhibitor (PD153035), 5 µM MEK–ERK inhibitor (U0126), 5 µM PI3K–AKT inhibitor (LY294002) or solvent [0.1% (v/v) DMSO] alone (Control). On day 1, the pathway inhibitor (or solvent) was replenished and, for some cultures, the medium was also supplemented with 10 µM GSK3β inhibitor (SB415286). Cell proliferation was determined using the MTT assay on days 0, 1, 4 and 6, with the respective culture medium in each case replenished on day 3. Data show the mean (±s.d.) absorbance at 570 nm (six technical replicates, three independent experiments); *P<0.05 when comparing the growth of cells treated with GSK3β inhibitor (SB415286) in the presence of EGFR inhibitor (PD153035) versus that of cells treated with PD153035 alone.

The observation that an EGFR–ERK-driven β-catenin–TCF pathway dominated in proliferative NHU cells (Fig. 2), combined with our finding that Wnt activation did not promote TCF activity over the high baseline (Fig. 1), led us to hypothesise that a blockade of EGFR would permit the influence of endogenous/exogenous mediators of β-catenin signalling to be observed. In EGFR-blocked NHU cell cultures, pharmacological activation of Wnt signalling using the GSK3β antagonist SB415286 resulted in intense punctate active β-catenin localisation in the nucleus (Fig. 3A). A comparison of mean nuclear fluorescence intensities showed that induction of Wnt signalling in EGFR-blocked cells restored nuclear active β-catenin levels to those observed in control EGFR-responsive NHU cells (Fig. 3A, right panel). The activation of Wnt signalling in NHU cells treated with the ERK inhibitor U0126 did not result in any profound changes in the expression or localisation of active β-catenin, and β-catenin localisation was more diffuse across both nuclear and cytoplasmic compartments (Fig. 3A). Moreover, in a large proportion of cells, active β-catenin labelling was also evident at the cell membrane (Fig. 3A). Inhibition of PI3K–AKT signalling resulted in a major increase in nuclear active β-catenin expression in comparison to controls (in agreement with Fig. 2), and activation of Wnt signalling by SB415286 had little effect on the overall intensity of active β-catenin expression, although the localisation appeared more diffuse (Fig. 3A).

The blockade of EGFR in NHU cells by PD153035 reduced basal TCF promoter activity, as was reflected by low levels of luciferase reporter activity in the TOPFLASH assays. This allowed for the first time the detection of significant induction of TCF promoter activity and, upon Wnt activation with SB415286, there was an approximately threefold increase in TOPFLASH (but not FOPFLASH) reporter activity (Fig. 3B), signifying substantial TCF promoter activation. Wnt-β-catenin activation in NHU cells treated with the MEK-ERK inhibitor U0126 had little effect on reporter activity (Fig. 3B). By contrast, reporter assays in PI3K–AKT-blocked NHU cells showed the previously observed (Fig. 1B) high basal TCF reporter activity, and GSK3β inhibition showed a modest yet non-significant increase in promoter activity (Fig. 3B)

The functional blockade of EGFR severely diminished NHU cell proliferation, but this could in part be overcome by pharmacological activation of Wnt signalling (Fig. 3C). Wnt stimulation in MEK–ERK-blocked NHU cells caused a small yet consistent increase in cell growth that was, however, not statistically significant (Fig. 3C). Inhibition of the PI3K–AKT pathway resulted in an initial retardation of NHU cell proliferation, consistent with our previous findings (MacLaine et al., 2008). However, Wnt activation had little effect on proliferation in PI3K–AKT-blocked NHU cell cultures (Fig. 3C).

### β-catenin modulates ERK and AKT signalling as part of a bi-directional positive-feedback signalling loop

**Fig. 4.**
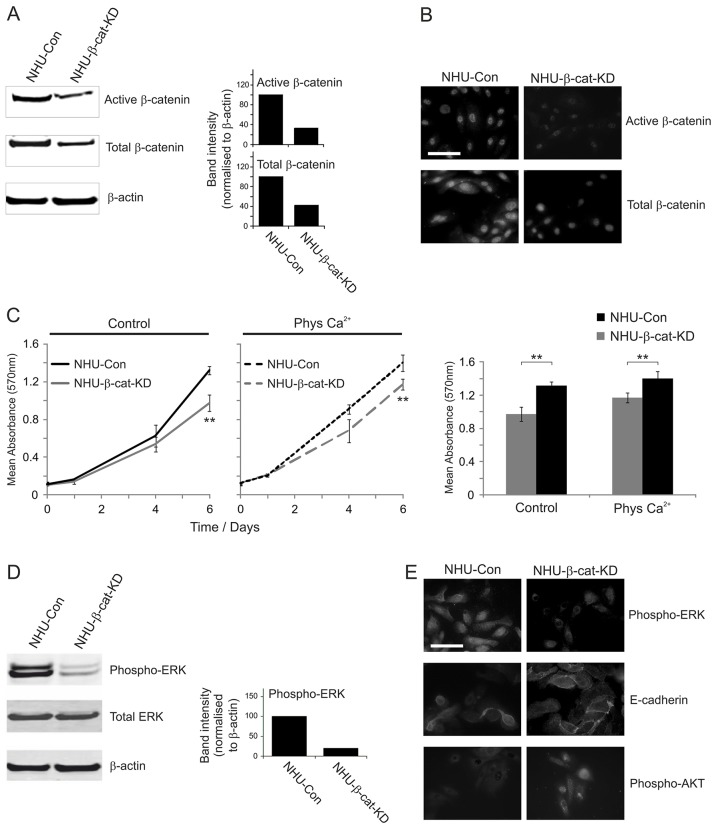
**The effect of RNAi-mediated β-catenin knockdown on EGFR–ERK and PI3K–AKT signalling activation and proliferation in NHU cells.** (A) Left, whole-cell lysates were prepared from NHU derivatives expressing β-catenin-specific shRNA (NHU-β-cat-KD) and their isogenic control-shRNA-expressing (NHU-Con) cells, and the expression of active and total β-catenin by was assessed by western blotting. β-actin served as a loading control. The results are representative of at least two experiments. Right, for the blots shown, β-catenin expression was quantified by densitometric analysis. Data show the relative band intensity of active and total β-catenin for NHU-β-cat-KD and NHU-Con cells after background subtraction and following normalisation to β-actin band intensity (the value for NHU-Con cells was arbitrarily set as 100). (B) Active and total β-catenin protein expression was detected by immunofluorescence microscopy in NHU-β-cat-KD and NHU-Con cells. The results are representative of at least three independent experiments. (C) NHU-β-cat-KD and NHU-Con cells were seeded (at 2×10^3^ cells/well) onto 96-well plates and allowed to attach overnight. Cells were cultured in either standard growth medium (Control) or medium supplemented with 2 mM CaCl_2_ (Phys Ca^2+^). Left, MTT assays were performed as for Fig. 3C. Data show the mean (±s.d.) absorbance at 570 nm (six technical replicates, three independent experiments). Right, Transformed data for results obtained on day 6 are shown. **P<0.01 when comparing the growth of NHU-β-cat-KD versus NHU-Con cells cultured in either standard growth medium or medium supplemented with 2 mM CaCl_2_. (D) Left, whole-cell lysates were prepared and immunoblotted as described in A for detection of phospho- and total ERK. β-actin served as a loading control. Results are representative of at least two independent experiments. Right, For the blots shown, phospho-ERK expression was quantified by densitometric analysis. Data show the relative band intensity of phospho-ERK for NHU-Con and NHU-β-cat-KD cells after background subtraction and following normalisation to β-actin band intensity (the value for NHU-Con cells was arbitrarily set as 100). Similar results were obtained when phospho-ERK was normalised to total ERK (not shown). (E) Total or phospho-ERK, phospho-AKT and E-cadherin protein expression was examined by immunofluorescence microscopy in NHU-β-cat-KD and NHU-Con cells. Results are representative of at least two independent experiments. Scale bars: 50 µm.

To investigate the role of β-catenin itself, we used a retroviral shRNA approach to generate NHU sub-lines carrying a stable β-catenin knockdown (NHU-β-cat-KD cells). Immunoblotting confirmed a substantial decrease in the expression of both active and total β-catenin in NHU-β-cat-KD cells in comparison with NHU-Con control-shRNA-expressing isogenic counterparts (Fig. 4A). Immunofluorescence microscopy confirmed these findings and showed that both nuclear active and total β-catenin expression were reduced in NHU-β-cat-KD cultures in comparison with controls (Fig. 4B).

NHU-β-cat-KD cell cultures showed a significant reduction in growth compared with NHU-Con cells over a 6-day time-course (Fig. 4C), which was suggestive of a crucial role for β-catenin activation in NHU cell proliferation. These results were supported by the finding that, in subconfluent cultures, fewer NHU-β-cat-KD cells appeared to be actively dividing, as indicated by a decreased number of Ki67-positive cells (not shown). Strikingly, β-catenin knockdown resulted in marked attenuation of active (phospho-ERK) but not total ERK expression (Fig. 4D,E). By contrast, loss of β-catenin enhanced E-cadherin expression and resulted in a dramatic increase in nuclear phospho-AKT (Fig. 4E).

### Activation and nuclear localisation of β-catenin is independent of the strength of cell–cell contacts

**Fig. 5.**
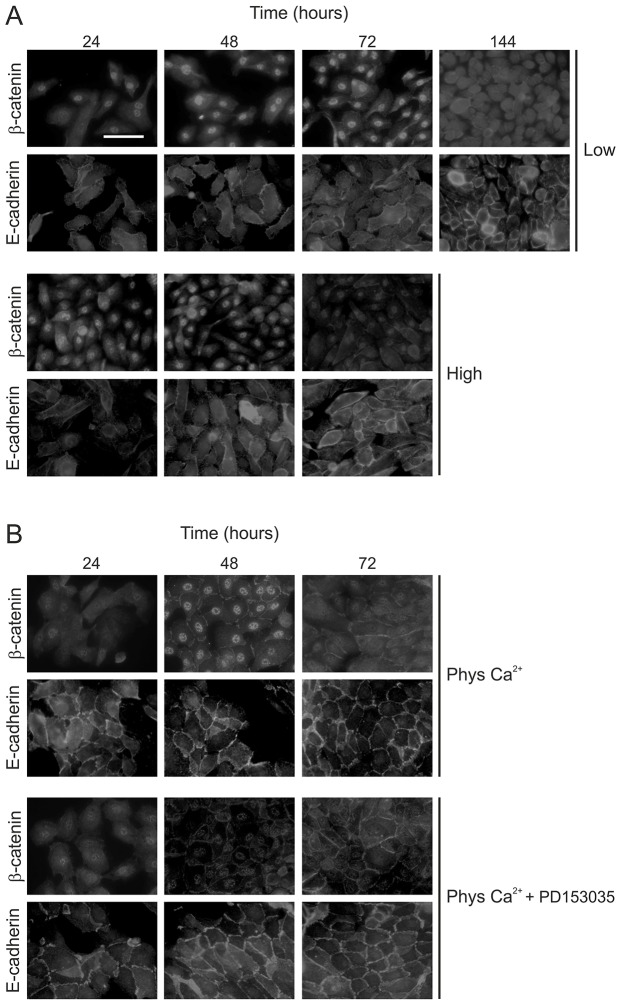
**The effect of culture density and calcium-mediated cell–cell contacts on the activation and localisation of β-catenin.** (A) NHU cells were seeded at 1.25×10^4^ cells/cm^2^ (Low) or 5×10^4^ cells/cm^2^ (High) and cultured for a total of 24, 48 or 72 hours (and 144 hours for low-density cultures) in standard culture medium. At the indicated time-points, cells were fixed and immunolabelled with antibodies for detection of active β-catenin and E-cadherin. (B) NHU cells were seeded at standard density (2.5×10^4^ cells/cm^2^) and cultured for a period of 24, 48 or 72 hours in medium supplemented with 2 mM CaCl_2_ (Phys Ca^2+^) in the presence or absence of 1 µM EGFR inhibitor (PD153035). Expression of active β-catenin and E-cadherin at the indicated time-points was detected as in A. Results are representative of at least three independent experiments. Scale bar: 50 µM.

Because of the fluctuation in nuclear expression of active β-catenin in NHU cell cultures over time, we altered the initial seeding density to assess whether this was an effect of culture density. In comparison with cultures seeded at 2.5×10^4^ cells/cm^2^ (Fig. 2A), seeding NHU cells at half this density (Fig. 5A, see figure legend) extended the length of time for which active β-catenin was present in the nucleus to at least 72 hours. A dramatic reduction in the nuclear β-catenin pool was evident at 144 hours post-seeding as cultures approached confluence (Fig. 5A). Seeding cells at a higher density did not ablate the initial nuclear translocation of active β-catenin, but the expression of active β-catenin diminished more rapidly (Fig. 5A).

The formation of adherens junctions has been shown to modulate the availability of β-catenin for nuclear translocation by sequestering the protein at the cell membrane (Nelson and Nusse, 2004). We thus sought to determine whether the quality and/or strength of cell–cell contacts could determine the localisation of active β-catenin. We used a Ca^2+^-switch approach, whereby increasing the exogenous Ca^2+^ concentration from 0.09 mM to a nearly physiological concentration (2 mM) results in the formation of stable E-cadherin-mediated cell–cell contacts (Georgopoulos et al., 2010). Increased Ca^2+^ concentration stimulated the formation of adherens junctions, and, by 48 hours, E-cadherin and some β-catenin was visible at the membrane (Fig. 5B). In common with the pattern observed under low Ca^2+^ conditions (Fig. 2A), strong active β-catenin expression was detectable in the nucleus at 48 hours post-seeding, although active β-catenin expression was lost by 72 hours.

Our previous studies have reported that progressive increase in cell density is associated with downregulation of EGFR–ERK signalling in NHU cultures (Georgopoulos et al., 2010). EGFR–ERK blockade with the EGFR inhibitor PD153035 not only severely reduced nuclear β-catenin localisation, but also increased the amount of active β-catenin at sites of cell–cell contact (Fig. 5B). The above observations were corroborated by western blotting, which revealed a strong (∼2.5-fold) increase in active β-catenin between the 24-hour and 48-hour time-points, which coincided with peak phospho-ERK levels and an ∼11-fold increase in inactive phospho-GSK3β (supplementary material Fig. S3). Notably, not only were strong cell contacts per se unable to sequester active β-catenin to the membrane, but they also did not alter the functional involvement of β-catenin in NHU cell proliferation as shown by Ca^2+^-switch experiments in control and NHU-β-cat-KD cells (Fig. 4C).

### Paracrine activation of Wnt–β-catenin signalling in NHU cells using exogenous Wnt ligands

We investigated whether exogenous Wnt ligand could activate canonical Wnt signalling in a paracrine fashion in NHU cells to regulate urothelial regeneration. We tested the effect of two independent ligands, Wnt3a and Wnt5a, which were chosen based on their identification as potential autocrine-expressed Wnt ligands in our gene expression analysis (above). Wnt3a has been previously shown to interact with several Fzd receptors, including Fzd2 and Fzd6, both expressed as transcripts by proliferating NHU cell cultures. Wnt5a has been shown to act as both an inhibitor and an activator of the canonical Wnt pathway, depending on receptor availability; if Fzd4 is present, Wnt5a can activate canonical signalling; however, if the tyrosine kinase ROR2 is present, Wnt5a inhibits the canonical Wnt cascade (Carmon and Loose, 2008). Affymetrix™ analysis indicated that ROR2 was absent, but that Fzd4 mRNA was present in proliferating NHU cultures and, in light of the high Wnt5a mRNA expression levels detected in proliferating NHU cultures, we anticipated that Wnt5a-driven canonical signalling should prevail.

**Fig. 6.**
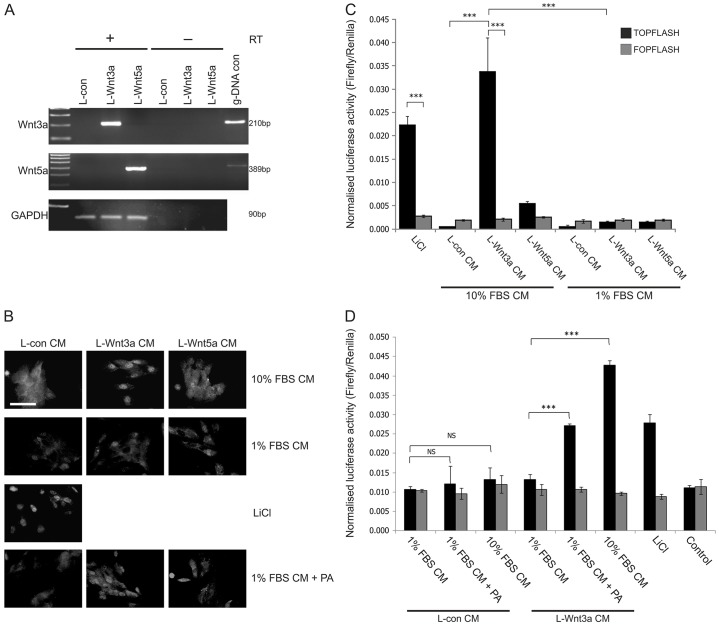
**Activation of Wnt–β-catenin signalling in SaOS-2 cells by treatment with Wnt3a- and Wnt5a-ligand-containing conditioned medium.** (A) Wnt3a and Wnt5a secreting L cells (L-Wnt3a and L-Wnt5a, respectively) and the parental control L cell line (L-Con) were tested by RT-PCR for expression of the respective Wnt ligand mRNA. PCR products were resolved by 1% (w/v) agarose gel electrophoresis and visualised using UV trans-illumination. Genomic DNA (g-DNA con) was used as a template control; GAPDH expression served as a loading control and to verify the presence of intact cDNA. Reverse transcriptase (RT)-negative (RT−) samples were included to prove the absence of g-DNA contamination. (B) Active β-catenin expression was detected by indirect immunofluorescence microscopy in SaOS-2 cells after a 24-hour treatment with conditioned medium (CM) from control, L-Wnt3a and L-Wnt5a cells (L-Con CM, L-Wnt3a CM and L-Wnt5a CM, respectively), as detailed in Materials and Methods. Conditioned medium was obtained from L cells cultured in standard medium supplemented with 10% (v/v) serum (10% FBS CM), medium with 1% (v/v) serum (1% FBS CM) and medium containing 1% (v/v) serum supplemented with 80 µM palmitic acid (1% FBS CM+PA). Treatment with L-con CM supplemented with 20 mM LiCl served as a positive control. Results are representative of three experiments. Scale bar: 50 µm. (C) SaOS-2 cells were transfected with either TOPFLASH or FOPFLASH plasmid along with transfection control pRL-CMV vector. At 24 hours post-transfection, cells were treated with L-Con CM, L-Wnt3a CM and L-Wnt5a CM, obtained from L cells cultured in medium supplemented with 10% (v/v) serum or 1% (v/v) serum. After 24 hours, dual luciferase reporter assays were performed. Data show the mean (±s.d.) firefly luciferase activity (three or four technical replicates following normalisation to transfection control, three independent experiments) (D) SaOS-2 cells were transfected as described for C and, 24 hours later, were treated with L-Con CM or L-Wnt3a CM that was obtained from L-Con and L-Wnt3a cells cultured in medium supplemented with 1% (v/v) serum, medium containing 1% (v/v) serum supplemented with 80 µM palmitic acid or medium supplemented with 10% (v/v) serum. After 24 hours, dual luciferase reporter assays were performed as for C. Treatment with 20 mM LiCl served as a positive control and negative controls included solvent-balanced conditioned medium [containing 0.1% (v/v) ethanol – not shown] and transfection alone with no treatment (Control). ***P<0.001; NS, non-significant.

Wnt3a and Wnt5a ligands were obtained from engineered L cell derivatives that actively secrete high levels of soluble ligand into the culture medium (Willert et al., 2003; Willert, 2008). We first confirmed that the L-Wnt3a and L-Wnt5a cells expressed the appropriate ligand by RT-PCR (Fig. 6A) and then verified their functionality using the Wnt-responsive SaOS-2 cells. To activate Wnt signalling, conditioned medium from each L cell line (cultured in medium containing 10% FBS) was harvested. Treatment with L-Wnt3a- but not L-Wnt5a-conditioned medium led to nuclear active β-catenin that was equivalent in intensity to that observed following LiCl treatment (Fig. 6B, top row); concordantly, activation of Wnt signalling resulted in an increase in TCF promoter activity (Fig. 6C).

**Fig. 7.**
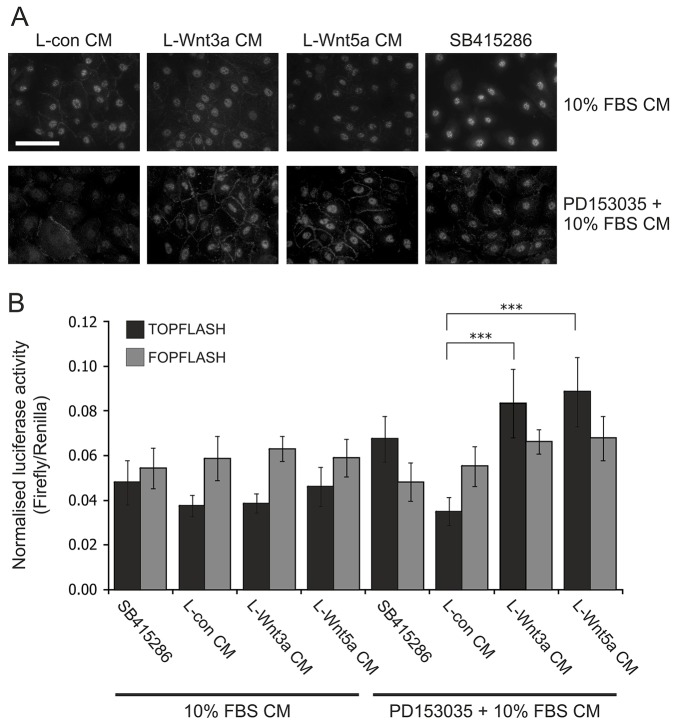
**Paracrine Wnt–β-catenin signalling activation in NHU cells: treatment with exogenous Wnt3a and Wnt5a ligands in EGF-responsive and EGFR-blocked cells.** (A) To detect active β-catenin protein expression by indirect immunofluorescence microscopy, NHU cells were pre-treated for 24 hours in the presence or absence of 1 µM EGFR inhibitor (PD153035). This was followed by a 24-hour treatment with conditioned medium (CM) from control (L-con), L-Wnt3a and L-Wnt5a cells (L-Con CM, L-Wnt3a CM and L-Wnt5a CM, respectively) obtained from L cells cultured in medium supplemented with 10% (v/v) serum. These treatments were carried out in the absence (10% FBS CM) or presence (PD153035+10% FBS CM) of EGFR inhibitor. Parallel cultures additionally treated with 10 µM GSK3β inhibitor (SB415286) were used as positive controls for β-catenin nuclear translocation. Results are representative of three experiments. Scale bar: 50 µm. (B) NHU cells were transfected with either TOPFLASH or FOPFLASH plasmid along with transfection control pRL-CMV vector. Starting at 16 hours post-transfection, cells were pre-treated with or without 1 µM EGFR inhibitor (PD153035) for 24 hours. Following this, cells were cultured in L-Con CM, L-Wnt3a CM or L-Wnt5a CM obtained from L cells cultured in medium supplemented with 10% (v/v) serum. These treatments were performed in the absence or presence of EGFR inhibitor. At 24 hours post-treatment, dual luciferase assays were performed. Data show the mean (±s.d.) firefly luciferase activity (three or four technical replicates following normalisation to transfection control, three independent experiments). ***P<0.001.

Addition of conditioned medium from all three L cell lines led to an increase in the amount of active β-catenin present at NHU cell–cell contact points (Fig. 7A), indicative of adherens junction formation due to the Ca^2+^ concentration of the DMEM (1.8 mM). In EGFR-blocked NHU cultures, incubation with either L-Wnt3a- or L-Wnt5a-conditioned medium resulted in strong nuclear active β-catenin localisation that was as intense as that observed in the presence of the GSK3β antagonist (Fig. 7A, lower panels). Accordingly, a significant increase in TCF reporter activity was observed when NHU cells were pre-treated and maintained in EGFR inhibitor and incubated with either L-Wnt3a- or L-Wnt5a-conditioned medium compared with L-con-conditioned medium (Fig. 7B). This demonstrated that NHU cells could respond canonically to both exogenous Wnt3a and Wnt5a ligands, but this was only evident following blockade of crosstalk from the EGFR signalling cascade.

### NHU cultures are capable of autocrine/paracrine Wnt-ligand-mediated activation and nuclear localisation of β-catenin

Because the addition of serum to NHU cell cultures will affect the proliferative phenotype and trigger differentiation (Cross et al., 2005), it was important to provide exogenous Wnt ligand to NHU cells in conditioned medium containing minimal serum. However, adaptation to 1% FBS resulted in L-cell derivatives failing to produce significant amounts of bioactive Wnt ligand when assessed in SaOS-2 cells (Fig. 6C). As palmitoylation is an essential post-translational modification for the biological activity of secreted Wnt ligands (Komekado et al., 2007; Willert et al., 2003), we titrated palmitic acid as a supplement in L cell cultures grown in 1% FBS (supplementary material Fig. S4). Supplementation of L-cell derivative cultures with 80 µM palmitic acid restored the functionality of secreted Wnt ligand, as evidenced both by the clear induction of nuclear β-catenin expression in SaOS-2 cells (Fig. 6B, lower panels) and by equivalent TCF promoter activity to that observed in SaOS-2 cells treated with LiCl (Fig. 6D).

**Fig. 8.**
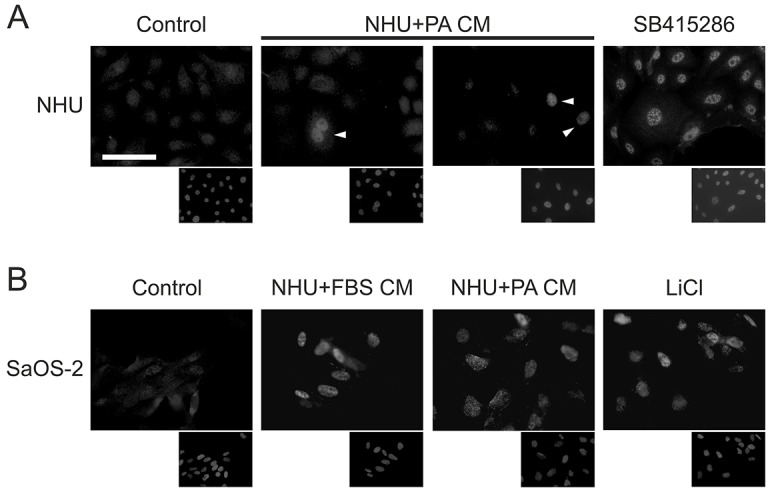
**Autocrine Wnt–β-catenin signalling in NHU cells.** (A) NHU cells were pre-treated for 24 hours with standard medium (KSFMc) supplemented with 1 µM EGFR inhibitor (PD153035), before treatment for 24 hours with conditioned medium (CM) from NHU cells cultured in KSFMc supplemented with 80 µM palmitic acid (NHU+PA CM). Expression of active β-catenin was detected by immunofluorescence microscopy. Parallel cultures treated with 10 µM GSK3β inhibitor (SB415286) were used as positive controls for β-catenin nuclear translocation. Negative controls included treatment with conditioned medium from NHU cells cultured in KSFMc containing 0.1% (v/v) ethanol alone (Control), as well as non-treated cells (not shown). Two representative images from one of three independent experiments are shown for NHU cells treated with conditioned medium (NHU+PA CM). White arrowheads, strong nuclear labelling in a subset of treated NHU cells. (B) SaOS-2 cells were treated for 24 hours with conditioned medium from NHU cells cultured in standard medium (KSFMc) in the presence of 10% (v/v) serum (NHU+FBS CM) or conditioned medium from NHU cells cultured in standard medium supplemented with 80 µM palmitic acid. Expression of active β-catenin was detected by immunofluorescence microscopy. Treatment with 20 mM LiCl served as a positive control for β-catenin activation. Negative controls were as described in A. For both A and B, lower panels show Hoechst 33258 labelling of nuclei. Scale bar: 50 µm.

As the secretion of bioactive Wnt from L cells was only achieved in the presence of serum or palmitic acid, the NHU cells grown in standard keratinocyte serum-free medium (KSFM) were unlikely to produce bioactive Wnt ligand. NHU cell cultures had been shown above to express Wnt3, Wnt5a, Wnt6 and Wnt7a transcripts, and to determine whether NHU cells had the potential to secrete functional Wnt ligand that could be used to drive canonical Wnt signalling in an autocrine/paracrine fashion, we prepared conditioned medium from NHU cells cultured in medium supplemented with either serum (NHU+FBS CM) or palmitic acid (NHU+PA CM). We initially tested these conditioned media for biological activity in SaOS-2 cells, where treatment with either NHU-derived conditioned medium resulted in nuclear translocation of active β-catenin, which was nearly as intense as that observed in cells treated with LiCl (Fig. 8B). By contrast, conditioned medium from control NHU cell cultures did not evoke any nuclear translocation of β-catenin in SaOS-2 cells (Fig. 8B, Control).

The effect of NHU+PA CM was tested on parallel homologous NHU cultures that were pre-treated with PD153035 inhibitor to block EGFR signalling; this was compared with vehicle and SB415286-treated control cultures. Treatment of EGFR-blocked NHU cells with NHU+PA CM did not result in population-wide nuclear active β-catenin (Fig. 8A), or any increase in TCF promoter activity (not shown). Strikingly, however, there was a consistent sub-population of cells that showed intense nuclear active β-catenin (Fig. 8A). Quantification of the immunofluorescence microscopy images showed that some 5% of cells were positive for nuclear active β-catenin. Similar results were obtained with NHU+FBS CM (not shown).

## DISCUSSION

The capacity of Wnt and β-catenin to crosstalk with and modulate the function of the MAPK pathways and, conversely, the capacity of RTKs to trigger β-catenin signalling are characteristics that are generally associated with cancer-derived cell lines where, by definition, there is dysregulation of normal homeostatic growth regulatory pathways. Using an epithelial cell culture system that retains normal features by being both of finite lifespan and competent to undergo differentiation, we have shown here that bidirectional interactions between Wnt–β-catenin and RTK–MAPK pathways operate contextually within a single epithelial cell type to regulate proliferation.

In conditions that inhibit the formation of stable adherens junctions, NHU cell cultures showed intense nuclear β-catenin staining associated with rapid population growth. We found a correlation between active β-catenin expression, ERK activity (phospho-42/44) and inactive (Ser9 phosphorylated) GSK3β, whereas blockade of EGFR or MAPKs reduced the expression and/or nuclear translocation of active β-catenin, ablated phospho-GSK3β and impaired TCF-mediated transcription. This crosstalk from MAPKs to β-catenin appears to be mediated through the phosphorylation of GSK3β and potentially by the ERK-mediated activation of the ribosomal proteins p70 S6 (officially known as RPS6KB2) and p90 RSK (officially known as RPS6KA1), as described in a variety of tumour cells (Ding et al., 2005).

A functional Wnt–β-catenin pathway has not been previously demonstrated in NHU cells, but analysis of the transcriptome of proliferating cells revealed the potential for an intact signalling pathway, with the expression of all key Wnt pathway components and absence of antagonists. Further compelling evidence for Wnt pathway activity in proliferating NHU cells was obtained from detection of Axin2, a feedback repressor and hallmark target of canonical Wnt signalling (Jho et al., 2002; Li et al., 2010), along with other downstream gene targets including survivin (Hughes and Brady, 2005), Twist (Zhang et al., 2001), SKP2 (Tang et al., 2009) and cyclin D1 (Howe et al., 2003). By contrast, Wif1, a potent extracellular antagonist of Wnt signalling, was only upregulated in quiescent contact-inhibited NHU cell cultures (supplementary material Fig. S1). Wif1 is frequently hypermethylated in urothelial carcinoma (Urakami et al., 2006) and its knockdown increases the cell proliferation rate, probably through increased transcription of c-Myc (Tang et al., 2009). Our results imply a homeostatic role for Wif1 in tissue quiescence through the silencing of Wnt signalling.

Initial experiments to activate Wnt pharmacologically with SB415286 failed to elicit a significant response because of high basal β-catenin activity. Thus, activation with Wnt ligand neither led to further significant increases in already substantial basal TCF activity, nor did it mediate any further cell proliferation increase in already highly proliferative NHU cells. Blockade of EGFR by pre-treatment with PD153035 efficiently inhibited all endogenous β-catenin activity and thence enabled SB415286 to influence β-catenin nuclear translocation and TCF-mediated transcription. This highlights not only a role for β-catenin crosstalk in mediating EGFR signalling in NHU cells, but also reveals that, in the absence of dominant EGFR signalling, alternative mechanisms of β-catenin activation are able to compensate to drive NHU cell proliferation. The fact that signalling downstream of EGFR must be completely blocked to observe a clear response with SB415286 implies that phosphorylation-mediated inhibition of GSK3β by MAPKs/ERKs might not be the only point of crosstalk between EGFR and β-catenin signalling cascades. The presence of a residual nuclear active β-catenin pool in NHU cells after treatment with U0126 indicates that a second convergence point between EGFR and β-catenin signalling pathways might exist. One such convergent point might be the EGFR tyrosine kinase itself – EGFR tyrosine kinase activity has previously been shown to phosphorylate β-catenin at Tyr654, destabilising the E-cadherin–β-catenin complex and releasing β-catenin for nuclear translocation (Kinch et al., 1995; Müller et al., 1999; Piedra et al., 2003).

Our results provide evidence that a bidirectional positive-feedback loop exists between Wnt–β-catenin and EGFR–ERK in NHU cells to efficiently drive sustained cellular proliferation. We have shown that EGFR-driven ERK-mediated inactivation of GSK3β results in β-catenin–TCF activation in NHU cells to promote proliferation. Blockade of EGFR–ERK (but not PI3K–AKT) signalling unmasked the potential for Wnt-pathway-mediated β-catenin activation, which, by triggering the activation of ERK, alleviated the ‘brake’ on proliferation exerted by EGFR blockade.

The knockdown of β-catenin (in NHU-β-cat-KD cells) caused significant reduction in NHU cell proliferation, resulting in a reduced proportion of NHU-β-cat-KD cells in cell cycle (based on Ki67 expression). β-catenin knockdown caused a significant reduction in phospho-ERK by an unknown mechanism, although it has been speculated that Raf1 is involved in activating ERK downstream of β-catenin (Yun et al., 2005). As a result of β-catenin knockdown, E-cadherin and phospho-AKT expression were both increased. The latter result is in agreement with our previous findings that EGFR–ERK and AKT signalling pathways are mutually exclusive in driving NHU cell proliferation (Georgopoulos et al., 2010). Thus, following the formation of stable adherens junctions (Georgopoulos et al., 2010) or β-catenin knockdown (here), NHU cells switch from a predominantly ERK-driven mode of proliferation to one that utilises the PI3K pathway. We have previously shown that NHU cell cultures treated with the PI3K antagonist LY294002 incur a short-lived inhibition of proliferation that is overcome within days (MacLaine et al., 2008), most probably as a result of the downregulation of E-cadherin, leading to the release of β-catenin to the nucleus. Here, growth assays performed in the presence of LY294002 revealed that NHU-β-cat-KD cells are more dependent on the PI3K–AKT pathway. Thus, NHU cells have the machinery to adapt and switch between the two growth-regulatory pathways. In one, EGFR signalling involves the activation of β-catenin, which, in turn, acts as a suppressor of the second, contact-dependent PI3K–AKT pathway.

Under adherens-junction-promoting conditions, active β-catenin is sequestered at intercellular junctions, resulting in the downregulation of β-catenin-regulated gene expression and increased cell–cell adhesion. Such sequestration of β-catenin is antagonised by a number of growth factor signalling mechanisms that either disrupt the adherens junction or downregulate E-cadherin expression and lead to a reduction in cell–cell contact coupled with an increase in TCF-mediated gene transcription. In this manner, β-catenin acts as a key coordinator of growth-factor-induced proliferation and cell–cell adhesion (Nelson and Nusse, 2004). When bound as an intracellular component of the adherens junction, β-catenin is spatially separated from the soluble cytoplasmic pool and is unable to translocate to the nucleus. In subconfluent cultures, β-catenin is found to be Tyr-phosphorylated at its C-terminal domain and does not interact with the components of the adherens junction (Müller et al., 1999; Shibamoto et al., 1994; Takeichi, 1995). Examples of Tyr-phosphorylation events that disrupt the adherens junction include phosphorylation of β-catenin at Tyr654 by Src or EGFR (disrupting the cadherin–β-catenin complex) and Tyr142 phosphorylation by Fer or Fyn (abrogating the interaction of β-catenin with α-catenin) (Kinch et al., 1995; Müller et al., 1999; Piedra et al., 2001; Piedra et al., 2003; Roura et al., 1999; Takeichi, 1995). By contrast, confluent cultures mainly express non-Tyr-phosphorylated, Ser/Thr-phosphorylated β-catenin, which localises at the membrane as an intrinsic component of the adherens junction (Müller et al., 1999; Shibamoto et al., 1994; Takeichi, 1995). Also, long-term EGF exposure can enhance E-cadherin repression by β-catenin–TCF-mediated expression of the transcriptional repressors Snail and Twist, suggesting a positive-feedback mechanism between the two pathways (Lickert et al., 2000), which is in agreement with our observation made here of Twist expression in proliferative NHU cells where β-catenin is actively signalling.

**Fig. 9.**
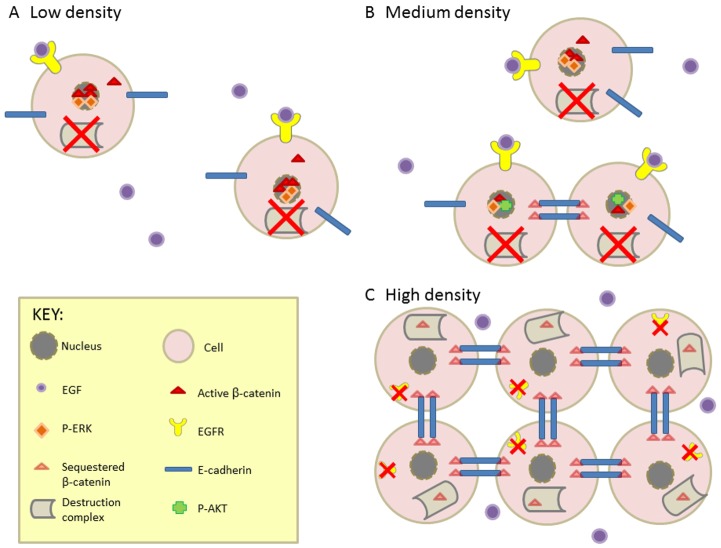
**Model of Wnt–β-catenin signalling crosstalk with EGFR–ERK and cell–cell-contact-mediated β-catenin regulation and their roles in NHU cell proliferation.** (A) At low density, EGFR present on the NHU cell surface is activated by EGF ligand. EGFR activation leads to phosphorylation of ERK (P-ERK), subsequent translocation of phospho-ERK to the nucleus and induction of cell proliferation. Phospho-ERK inactivates the destruction complex, allowing β-catenin to accumulate and enter the nucleus. (B) As culture density increases, the intense signalling crosstalk between β-catenin and EGFR–ERK results in a positive-feedback loop that accelerates growth and supports a highly proliferative (regenerative) phenotype, thus leading to rapid increases in cell numbers. Interestingly, the activities of β-catenin and AKT appear to be mutually exclusive. (C) Confluence-induced reduction in the expression of EGFR leads to a decline in EGFR signalling and reduction in phospho-inhibition of GSK3β. As a consequence, the activity of the destruction complex is reinstated. Moreover, cytosolic β-catenin is sequestered to the cytoplasmic tail of E-cadherin and, as a result, extensive adherens junctions form. Once a critical number of cell–cell contacts have been established, excess β-catenin is targeted for degradation by the destruction complex. Phospho-ERK and active β-catenin levels are low, cells enter G1 growth arrest and cells exit the regenerative response.

Seeding NHU cells at low density resulted in prolonged nuclear expression of β-catenin, whereas high seeding densities resulted in weak nuclear expression. The most obvious explanation for this apparent confluence-dependent effect on β-catenin activity is provided by our previously published findings showing EGFR downregulation in confluent NHU cell cultures (Varley et al., 2005). The present work corroborates these published findings, as a reduction in the expression of phospho-ERK was observed as NHU cultures became more densely populated. The ability of adherens junction formation to modulate the availability of β-catenin for nuclear translocation by sequestering the protein at the cell membrane could potentially be an important link in modulating the proliferation-quiescence switch in NHU cells. In low-Ca^2+^ growth medium, such as KSFMc, Ca^2+^-dependent cell–cell engagement is weak and β-catenin expression at the cell membrane is low. However, stimulating adherens junction engagement by increasing the concentration of extracellular Ca^2+^ did not reduce the levels of nuclear β-catenin or the activity of TCF. These results suggest that, once released from the destruction complex, β-catenin preferentially translocates to the nucleus. How this is regulated remains unclear, but evidence suggests that activated EGFR can phosphorylate β-catenin at Tyr654, negating its ability to interact with E-cadherin at the adherens junction (Kinch et al., 1995; Piedra et al., 2003). Our findings support this scenario, as blockade of EGFR not only reduced the level of nuclear β-catenin but also increased the amount of β-catenin at the membrane. Therefore, our observations demonstrate that the quality of cell–cell contacts, although important in increasing the level of active β-catenin sequestered at sites of contact, does not alter the ability of β-catenin to translocate to the nucleus, nor does it interfere with its function in epithelial growth. Instead, β-catenin activation is predominantly dictated by proliferative signalling cues, and its gradual downregulation in cultured cells coincides with progression from cell proliferation to quiescence. Collectively, our study suggests that β-catenin might be crucial in the proliferation-quiescence switch seen during tissue regeneration. Overall, there appears to be a complex communication network between EGFR signalling, Wnt–β-catenin signalling and cell–cell contact, as summarised in Fig. 9. We suggest that β-catenin plays a central role in the regulation of this network, which strengthens the already prominent role of β-catenin in the maintenance of normal epithelial tissue homeostasis and renders it a potential target for deregulation in the transition from normal to malignant cell growth.

In addition to pharmacological activation of Wnt signalling, we examined whether biologically active Wnt ligand family members could trigger canonical Wnt signalling in NHU cells. For this purpose, we utilised L-cell derivatives producing Wnt ligands in conditioned medium. Previous reports on the production of bioactive Wnt ligand have recommended the inclusion of 10% (v/v) serum when harvesting conditioned medium, as serum contains albumin-bound palmitic acid that is required for Wnt post-translational palmitoylation (Povelones and Nusse, 2005). However, this imposed a practical difficulty for studying exogenous Wnt signalling in NHU cell cultures and for assessing whether NHU cells were capable of producing autocrine Wnt ligand, as serum is known to induce NHU differentiation (Cross et al., 2005). Wnt ligands are hydrophobic in nature owing to the many essential lipid modifications that occur during the maturation process. Post-translational modification of Wnt occurs in the ER and begins with the addition of a hydrophobic palmitate moiety, a process known as palmitoylation, which occurs on the first, absolutely conserved cysteine residue (Cys77 in Wnt 3a and Cys104 in Wnt 5a) (Reichsman et al., 1996; Willert et al., 2003) and which is essential for activity but not for secretion itself (Komekado et al., 2007; Willert et al., 2003). In our study, conditioned medium from serum-reduced Wnt-secreting L cell lines did not produce significant amounts of bioactive Wnt ligand and thus supported the results presented in previous publications (Willert et al., 2003; Willert, 2008). To overcome this, palmitic acid was added to the growth medium of L Wnt-3a cells to replace serum. Because of issues with the solubility and precipitation of palmitic acid in aqueous medium (not shown) a maximum concentration of 80 µM palmitic acid was obtained (compared with 110 µM in medium containing 10% FBS, as measured by gas-liquid chromatography; not shown). This was adequate to restore Wnt ligand secretion and activity to ∼50% of that observed with serum-supplemented medium, thus confirming that palmitic acid was required for the production of bioactive Wnt ligand. This observation provides the first conclusive evidence that serum can be reduced when harvesting bioactive Wnt ligand, but only if the growth medium is supplemented with palmitic acid. More importantly, by exploiting the successful palmitic-acid-based approach to produce active Wnt ligands, we were able to demonstrate for the first time that biologically active Wnt ligands can induce canonical Wnt–β-catenin signalling in NHU cells.

Our studies also provided some intriguing observations when we examined the ability of NHU cells to carry out autocrine/paracrine Wnt signalling by endogenously produced Wnt ligands. We demonstrated that EGFR-blocked NHU cells that had been incubated with conditioned medium from isogenic palmitic-acid-treated NHU cell cultures showed limited overall Wnt pathway activation, which was restricted to a small proportion of NHU cells that displayed high levels of active nuclear β-catenin after treatment. At present, the basis for this heterogeneity and its implications are unclear; however, the data imply that there might be a small subset of cells capable of driving self-renewal in response to autocrine/paracrine Wnt signalling. It is tempting to speculate that this might represent a subpopulation of cells with the ability to initiate their own programme of self-renewal, such as a stem cell population.

The presence of resident adult stem cell populations has been described for many organs, including brain, lung and heart, as well as many epithelial tissues, including liver, colon and skin (Mimeault and Batra, 2008). Although research into urothelial stem cells is ongoing, no unequivocal resident stem cell population has been identified in human urothelium, although *in situ* observations have been used to infer a basal progenitor (Gaisa et al., 2011). In the rat, a subpopulation of highly clonogenic BrdU-label-retaining (i.e. long lived) basal cells have been identified and have been shown to express markers consistent with stem cells in other tissues (including Bcl-2, p63, KRT14 and β1 integrin) (Kurzrock et al., 2008). In the mouse, a subset of KRT5^+^ basal urothelial cells have been shown to express Sonic hedgehog (Shh), a ligand that is important during embryonic development. The relevance of these studies to human urothelium remains unclear, as there appear to be fundamental differences in the regulation of urothelial regeneration between human and rodent urothelium *in vivo* (Chopra et al., 2008), and the plasticity to revert from a suprabasal to a basal phenotype is a feature of human urothelial cells, at least *in vitro* (Wezel et al., 2013). The role of Wnt–β-catenin signalling has been more widely studied in rodent urothelium than in human, and the pathway has been found to play an important role in tissue homeostasis. In the mouse, proliferation in response to bacterially or chemically induced injury is regulated by signal feedback between the basal urothelial cells and the underlying stromal cells. After injury, basal urothelial cells were seen to secrete Shh, evoking the expression of Wnt ligands from the underlying stroma. Both stromal and urothelial cells proliferated in response to Wnt ligand, restoring urothelial integrity (Shin et al., 2011). Our observation of autocrine/paracrine Wnt–β-catenin activation in a subset of NHU cells might represent an important step towards identifying self-renewal mechanisms in human urothelium.

In summary, our study provides evidence for a bi-directional signalling loop between Wnt–β-catenin and RTK-driven MAPK signalling pathways that serves to drive proliferation in a normal epithelial cell population. This has important implications for normal epithelial physiology, where the crosstalk could represent an extremely efficient mechanism to rapidly initiate, accelerate and sustain cell growth during tissue regeneration, for instance, following tissue damage. Upon completion of tissue regeneration and establishment of contact inhibition, rapid cell-contact-mediated downregulation of RTK signalling (combined with the induction of inhibitory Wnt components) would attenuate β-catenin signalling, thus switching off the signalling feedback loop and, subsequently, cell proliferation. Moreover, this efficient mechanism would represent a molecular target in carcinogenesis, as its dysregulation (constitutive activation) would provide a strong growth advantage during malignant transformation (Ahmad et al., 2011a; Ahmad et al., 2011b).

## MATERIALS AND METHODS

### Reagents and antibodies

Pharmacological inhibitors PD153035, U0126 and LY294002 were purchased from VWR (Merck). GSK3β inhibitors SB415286 and LiCl were from Sigma Aldrich. The antibodies used were against; active β-catenin dephosphorylated on Ser37 and Thr41 (8E7; a kind gift from Hans Clevers, Utrecht University), total β-catenin (C2206; Sigma Aldrich), β-actin (AC-15; Sigma), E-cadherin (HECD-1; Abcam), total ERK (16; Transduction Laboratories), phospho-42/44 MAPK (D13.14.4E; Cell Signalling Technology), AKT (7; BD Biosciences), phospho-473 AKT (clone D9E; Cell Signalling Technology) and phospho-9 GSK3β (AB30619; Abcam). The secondary antibodies were from Invitrogen. The secondary antibodies for immunofluorescence microscopy were Alexa-Fluor-488-conjugated goat anti-mouse-IgG and Alexa-Fluor-594-conjugated goat anti-rabbit-IgG, and those used for western blotting were Alexa-Fluor-680-conjugated goat anti-mouse-IgG, Alexa-Fluor-800-conjugated goat anti-rabbit-IgG and Alexa-Fluor-680-conjugated donkey anti-goat-IgG.

### NHU cell culture

Surgical specimens of normal ureteric urothelium were obtained with NHS Research Ethics Committee approval and with informed consent from patients with no histological evidence of urothelial dysplasia or malignancy. The preparation and maintenance of finite NHU cell lines was as detailed previously (Southgate et al., 1994; Southgate et al., 2002), and NHU cultures were maintained in complete supplemented keratinocyte serum-free medium (KSFMc). In the studies described here, an assessment of cell death is not included as it did not play a contributory role.

### Cell lines

The β-catenin-knockdown shRNA-expressing sub-line (NHU-β-cat-KD) and its isogenic control-shRNA-expressing sub-line (NHU-Con) have been described previously (Georgopoulos et al., 2010). SaOS-2 cells were obtained from the ATCC and cultured in McCoy's 5A medium supplemented with 15% (v/v) FBS. L-Wnt3a, L-Wnt5a and parental L cells (Willert, 2008) were provided by Paul Genever (University of York) and were cultured in DMEM containing 10% (v/v) FBS. These lines were used for the production of Wnt-ligand-containing conditioned medium.

### Production of conditioned medium

Conditioned medium was collected from L-cell derivatives as described previously (Qiang et al., 2009) and filtered through 0.2-µm Tuffryn® filters (VWR) prior to immediate use or storage at 4°C. For some experiments, 3-day-conditioned medium was collected from L cell lines or NHU cells maintained in medium [DMEM with 1% (v/v) FBS or KSFMc, respectively] and supplemented with 80 µM palmitic acid (added to prewarmed medium from a 20 mg/ml stock in ethanol). Conditioned medium was tested for its ability to activate the canonical Wnt pathway in SaOS-2 and NHU cells by dilution 1∶10 (v/v) with standard growth medium for each target cell type (i.e. McCoy's 5A with 15% FBS or KSFMc, respectively).

### Affymetrix data analysis

Expression array data analysed in the present study were selected from an experimental series analysed using Affymetrix™ GeneChip Human Genome U133 Plus 2.0 (HG-U133 Plus 2.0) arrays, as described previously (Böck et al., 2014), and the data are available in the ArrayExpress database under the accession number E-MTAB-2188. Affymetrix .CEL files were analysed using Arrayassist™ 5.5.1 software (Agilent). Background was removed and chips were normalised using the Microarray suite 5 (MAS5) algorithm, before an absolute calls database was generated. The arrays representing non-differentiated NHU cell cultures over a time series of 6, 24, 72 and 144 hours were assessed for the expression of cell-cycle-associated MKI67 and the ‘proliferation signature’ genes PLK1, BUB1 and TOP2A (Tago et al., 2000). From this, the 24-hour and 144-hour cultures were selected for study as representing the most and least proliferative time-points, respectively. The data on the signal intensities for these two arrays were transferred to Excel® and Ingenuity® Pathway Analysis (IPA) software (Ingenuity Systems) for further analysis.

### Reverse transcription-PCR and real-time PCR analysis

Quantification of transcript expression was conducted by real-time PCR using SYBR® green reagents (Applied Biosystems) or by semi-quantitative reverse transcription (RT)-PCR. Forward and reverse target gene primer sequences are provided in the supplementary material Table S1. RNA was isolated, cDNA synthesised and PCR performed using protocols and reaction profiles described elsewhere (Fleming et al., 2012). Genomic DNA (gDNA) was used as the control for RT-PCR and was purified from cultured cells using a DNeasy Blood and Tissue Kit (Qiagen).

### Western blotting

Immunoblotting was performed as described elsewhere (Georgopoulos et al., 2010) and involved SDS-PAGE and protein transfer onto Immobilon-FL polyvinylidene fluoride (PVDF) membrane (Millipore) and probing with primary antibodies and then appropriate secondary antibodies. Immunolabelling was visualised on a LI-COR Odyssey™ infrared imaging system and band intensity was quantified by densitometric analysis using Odyssey software.

### Immunofluorescence microscopy

NHU and SaOS-2 cells were seeded at a density of 5×10^3^ to 2×10^4^ cells/well (depending on the experiment) on Teflon-coated 12-well glass slides. Cells were fixed either in a 1∶1 (v/v) mixture of methanol∶acetone or in 10% (v/v) formalin in PBS. Formalin-fixed slides were permeabilised in 0.1% (w/v) Triton X-100. Nuclei were visualised with 0.1 mg/ml Hoechst 33258, and proteins of interest were detected with the specific primary antibody followed by appropriate secondary conjugated to Alexa Fluor 488 or Alexa Fluor 594. Slides were examined under epi-fluorescence illumination on an Olympus BX60 microscope, and nuclear and cytoplasmic labelling were quantified using Photoshop (Adobe) as described previously (Hubbell et al., 2002). Briefly, Hoechst 33258 images were superimposed onto the adjacent fluorescent image to be quantified. Background labelling was normalised and the Hoechst 33258 image was used as a mask to calculate mean nuclear intensity from the histogram. The selection was then inversed to give cytoplasmic and membrane labelling intensities.

### Cell proliferation assays

Cell proliferation was indirectly determined by measurement of cell culture biomass using the MTT assay. Cells were seeded onto 96-well plates at a seeding density of 2×10^3^ cells/well (under low or physiological Ca^2+^ conditions for NHU cells) and were incubated for 24 hours at 37°C (day −1). Cells were treated with pharmacological inhibitors (single inhibitors or combinations thereof) in triplicate wells (day 0) and plates were assayed by MTT on days 0, 1, 3 and 6. A solvent balanced control was included at each time-point. Medium and inhibitors were replenished on day 3.

### Luciferase reporter assays

NHU and SaOS-2 cells were seeded onto 24-well plates at a density of 4×10^4^ cells/well. The following day, 0.5 µg of TOPFLASH or FOPFLASH vector (along with 0.01 µg of pRL-CMV plasmid) was transfected into NHU cells using Fugene® HD reagent (Promega). Transfected cells were incubated for 16 hours, the medium was replaced with normal growth medium and cells were treated with Wnt ligand or GSK3β inhibitor for 24–72 hours. Lysates were prepared and Dual Luciferase Assays were performed as recommended by the manufacturer (Promega).

### Statistical analysis

Statistics were performed using GraphPad InStat v3.05 (GraphPad). Parametric statistics [mean and s.d. (n−1)] were used for descriptive purposes, and tests of significance were by means of a two-tailed Student's *t*-test – significance was assumed when *P*≤0.05.

## Supplementary Material

Supplementary Material
