# Supplementary material for: A novel bidirectional positive-feedback loop between Wnt–β-catenin and EGFR–ERK plays a role in context-specific modulation of epithelial tissue regeneration
Source: J Cell Sci. 2014 Jul 1;127(13):2967–82. doi: 10.1242/jcs.150888 (PMC4077591; doi:10.1242/jcs.150888)
Supplement: Supplementary Material [file supp_127_13_2967__index.html]

A novel bidirectional positive-feedback loop between Wnt–β-catenin and EGFR–ERK plays a role in context-specific modulation of epithelial tissue regeneration — Supplementary Material 

# A novel bidirectional positive-feedback loop between Wnt–β-catenin and EGFR–ERK plays a role in context-specific modulation of epithelial tissue regeneration

## JCS150888 Supplementary Material

**Files in this Data Supplement:**

- **Supplementary Material**
