# Supplementary material for: A novel bidirectional positive-feedback loop between Wnt–β-catenin and EGFR–ERK plays a role in context-specific modulation of epithelial tissue regeneration
Source: J Cell Sci. 2014 Jul 1;127(13):2967–82. doi: 10.1242/jcs.150888 (PMC4077591; doi:10.1242/jcs.150888)
Supplement: Supplementary Material [file supp_127.13.2967_JCS150888.pdf]

Supplementary data: A novel bidirectional positive feedback loop between Wnt/β-catenin and EGFR/ERK: role of context-specific signalling crosstalk in modulating epithelial tissue regeneration

Supplementary Table 1

DNA sequences of forward (FWD) and reverse (REV) oligonucleotide primers used in this study.

Gene target	FWD 5′ → 3′	REV 5′ → 3′
**Axin2**	CAAGGGCCAGGTCACCAA	CCCCCAACCCATCTTCGT
**CDH1**	AATCTGAAAGCGGCTGATACTGA	CGGAACCGCTTCCTTCATAG
**GAPDH**	CAAGGTCATCCATGACAACTTTG	GGGCCATCCACAGTCTTCTG
**Ki67**	CAAGAGCATCAGAACGTTTAAGGA	TTCTTGGCCACTTCTTCATTCC
**Wif1**	GGCACCTTTTACACATGATTTCAG	TGGAATGGATATTGACAGGAATAGC
**Wnt3a (mouse)**	CTGGCAGCTGTGAAGTGAAG	TGGGTGAGGCCTCGTAGTAG
**Wnt5a (mouse)**	CTGGCTCCTGTAGCCTCAAG	AATCTCCGTGCACTTCTTGC

A

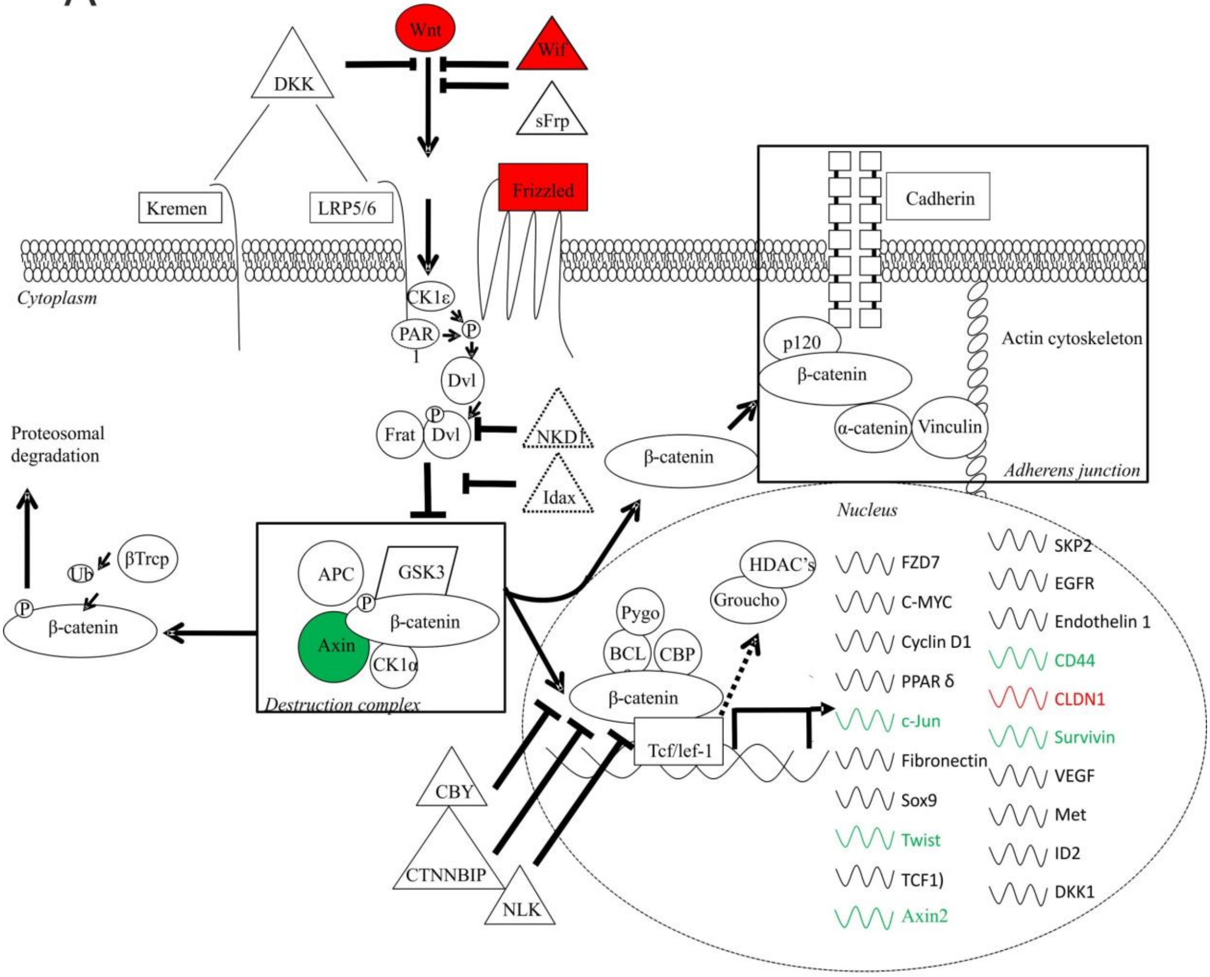
Wnt
Wif
DKK
sFrp
Kremen
LRP5/6
Frizzled
Cadherin
Cytoplasm
CK1ε
PAR
P
Dvl
Actin cytoskeleton
p120
β-catenin
Proteosomal degradation
Frat
Dvl
NKD1
α-catenin
Vinculin
β-catenin
Adherens junction
Idax
Nucleus
βTrcp
Ub
APC
GSK3
HDAC's
P
β-catenin
Groucho
β-catenin
Axin
Pygo
CK1α
BCL
CBP
Destruction complex
β-catenin
Tcf/lef-1
CBY
CTNNBIP
NLK
FZD7
C-MYC
Cyclin D1
PPAR δ
c-Jun
Fibronectin
Sox9
Twist
TCF1)
Axin2
SKP2
EGFR
Endothelin 1
CD44
CLDN1
Survivin
VEGF
Met
ID2
DKK1

B

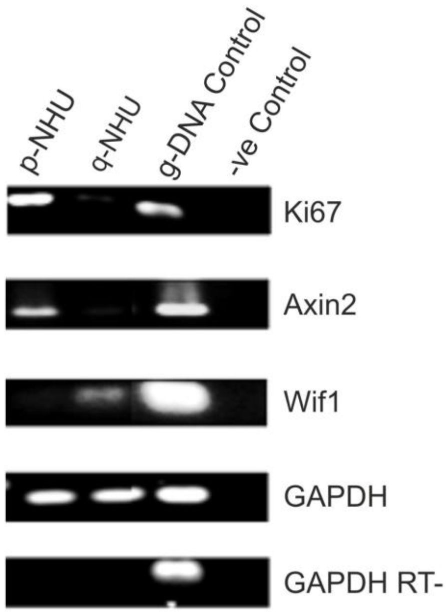
p-NHU
q-NHU
g-DNA Control
-ve Control
Ki67
Axin2
Wif1
GAPDH
GAPDH RT-

Supplementary Figure 1

Expression of Wnt/β-catenin pathway components in NHU cells assessed by expression arrays and RT-PCR

A) Schematic representation of the components of the canonical Wnt cascade present in NHU cells and gene changes relating to the pathway in quiescent cells. Wnt pathway components in the diagram (white or in colour) were classified as 'present' from MAS5 normalised Affymetrix^{TM} microarray data. Genes with at least a 2-fold change in expression in quiescent NHU cultures compared to proliferative cultures are labelled as red (up-regulated) or green (down-regulated) and fold-change gene expression data are provided in appropriate tables. Components in white with dashed borders were classed as absent in the proliferating NHU culture.

B) Expression of Wnt components in NHU cells grown in culture as a proliferative monolayer (p-NHU) and in fully-confluent, quiescent cultures (q-NHU) was assessed by RT-PCR (using primers the sequences of which are provided in Supplementary Table 1). Human genomic DNA (g-DNA) was used as a template control, whilst water only was used as a no-template negative control (-ve Control). GAPDH was used as a housekeeping control to verify intact cDNA. Reverse transcriptase (RT) negative samples (GAPDH RT-) were included to verify lack of genomic DNA contamination in the cDNA template.

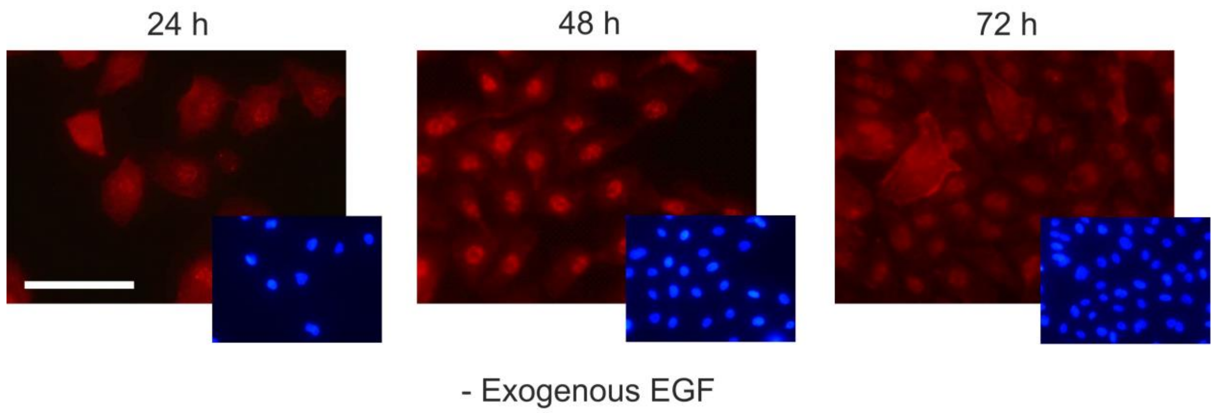

Supplementary Figure 2

Expression and localisation of active β-catenin in NHU cells cultured without exogenous recombinant human EGF (rhEGF)

NHU cells were cultured for 24, 48 and 72 hours in KSFM medium supplemented with BPE but in the absence of rhEGF ligand (-Exogenous EGF). Cells were formalin-fixed and immunolabelled with active β-catenin antibody (8E7) and rabbit anti-mouse IgG Alexa 594 secondary antibody. Cells were also stained with Hoechst 33258 to delineate the nucleus. Labelling was visualised under epifluorescence illumination. Scale bar: 50μm.

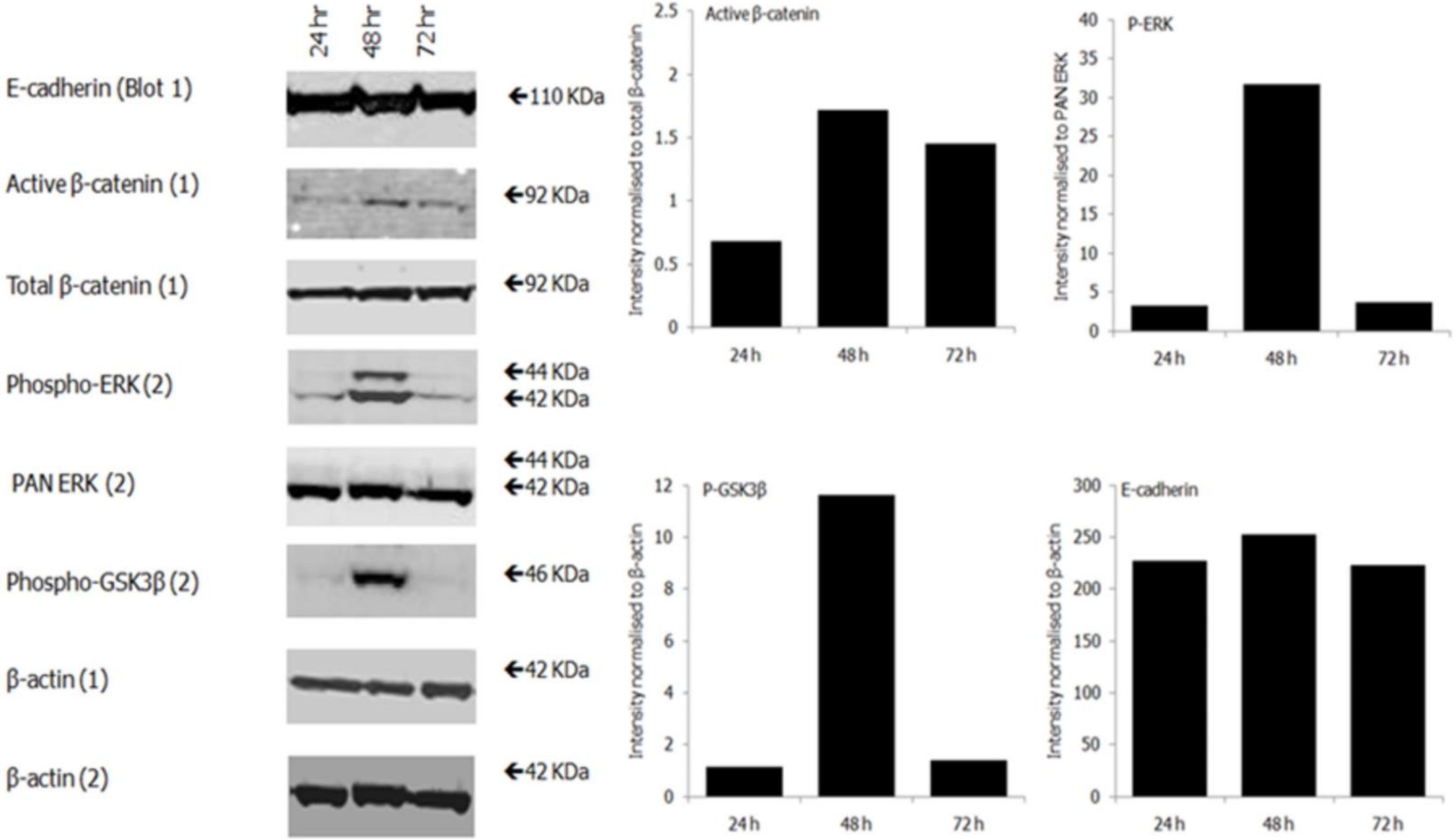
24 hr
48 hr
72 hr
E-cadherin (Blot 1)
←110 KDa
Active β-catenin (1)
←92 KDa
Total β-catenin (1)
←92 KDa
Phospho-ERK (2)
←44 KDa
←42 KDa
PAN ERK (2)
←44 KDa
←42 KDa
Phospho-GSK3β (2)
←46 KDa
β-actin (1)
←42 KDa
β-actin (2)
←42 KDa
Active β-catenin
Intensity normalised to total β-catenin
0
0.5
1
1.5
2
2.5
24 h
48 h
72 h
P-ERK
Intensity normalised to PAN ERK
0
5
10
15
20
25
30
35
40
24 h
48 h
72 h
P-GSK3β
Intensity normalised to β-actin
0
2
4
6
8
10
12
24 h
48 h
72 h
E-cadherin
Intensity normalised to β-actin
0
50
100
150
200
250
300
24 h
48 h
72 h

Supplementary Figure 3

Expression of Wnt/β-catenin pathway components in NHU cells over a 72 hour time-course in physiological calcium conditions

NHU cells were cultured as described in Figure 5B, before whole cell lysates were prepared and expression of E-cadherin, β-catenin (Active and Total), ERK (Phospho- and Total), and Phospho-GSK3β (Serine 9) was assessed by Western blotting. Detection of β-actin served as a loading control. Results (blots shown on the left) are representative of experiments with two NHU lines. For the blots shown, densitometric analysis was also carried out to quantify β-catenin expression. Bars (on the right) represent relative band intensity of active β-catenin, phospho-ERK, phospho-GSK3β and E-cadherin after background subtraction and following normalisation to respective β-actin band intensity. The appropriate blot number ("1" or "2") and its corresponding β-actin expression are indicated.

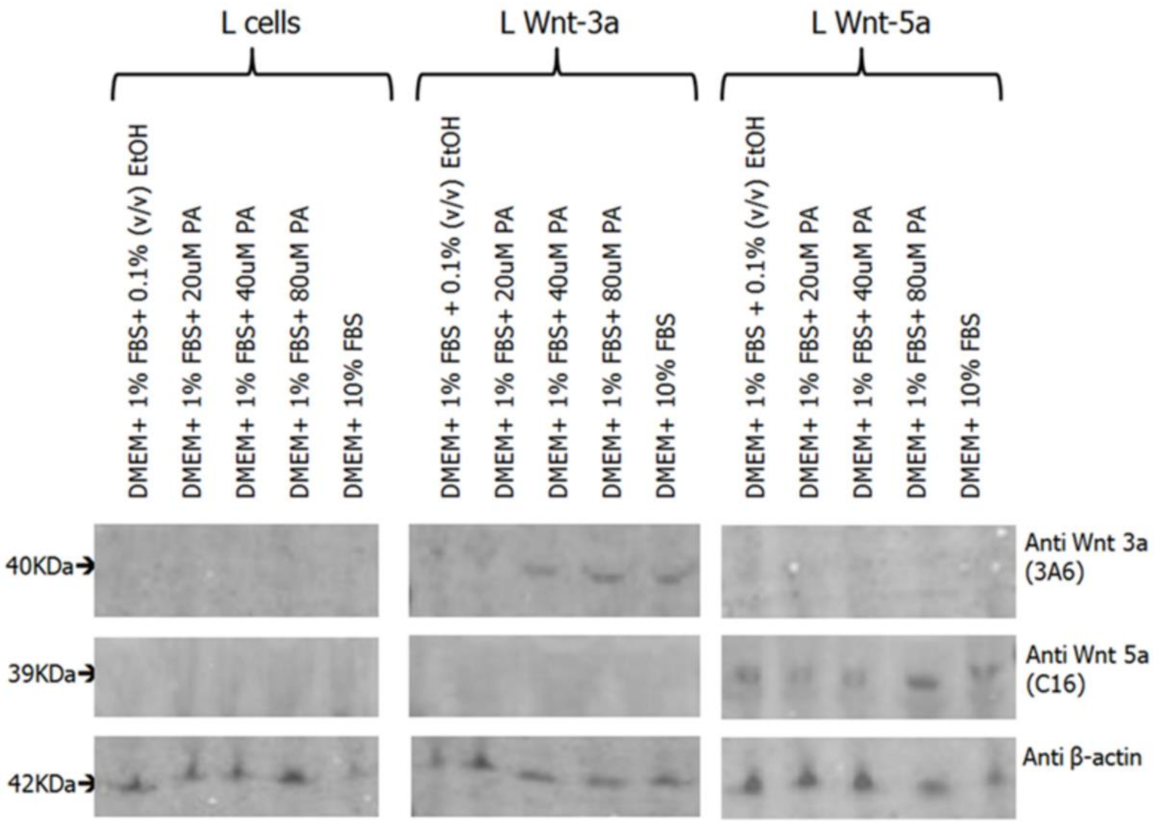

Supplementary Figure 4

Assessing the effect of palmitic acid (PA) on Wnt ligand production

Whole cell lysates were prepared from L-Wnt3a, L-Wnt5a and L-Con cells cultured for 72 hours in medium (DMEM) containing 1% FBS (v/v) and supplemented with 20-80μM palmitic acid (PA). Positive and negative controls were 10% (v/v) FBS and 0.1%(v/v) EtOH (solvent only), respectively. 20μg of each protein lysate was subjected to SDS-PAGE within a 4-12% (w/v) Bis-Tris gel under denaturing conditions and immunoblotted onto PVDF membrane. Membranes were probed with anti-Wnt 3a (mouse 3A6; Santa Cruz), anti-Wnt 5a (goat C16; Santa Cruz) and β-actin (AC-15; Sigma) antibodies followed by goat anti-mouse IgG Alexa 680 for Wnt 3a and β-actin or donkey anti-goat IgG Alexa 680 for Wnt-5a. Antibody binding was visualised as described in the Methods section.
